# Sodium Nitroprusside Improves the Growth and Behavior of the Stomata of *Silybum marianum* L. Subjected to Different Degrees of Drought

**DOI:** 10.3390/life13040875

**Published:** 2023-03-24

**Authors:** Esmaeil Zangani, Hossein Rabbi Angourani, Babak Andalibi, Saeid Vaezi Rad, Andrea Mastinu

**Affiliations:** 1 Department of Plant Production and Genetics, University of Zanjan, Zanjan 45371-38791, Iran; andalibi@znu.ac.ir; 2 Research Institute of Modern Biological Techniques, University of Zanjan, Zanjan 45371-38791, Iran; rabbihosein@znu.ac.ir; 3 Department of Agronomy, Science and Research Branch, Islamic Azad University, Zanjan 45156-58145, Iran; saeidvaezi@iauz.ac.ir; 4 Department of Molecular and Translational Medicine, Division of Pharmacology, University of Brescia, 25123 Brescia, Italy

**Keywords:** active constituents, grain yield, *Silybum marianum* L., nitric oxide, water stress

## Abstract

The use of growth-stimulating signals to increase the tolerance of plants to water deficits can be an important strategy in the production of plants in dry areas. Therefore, a split-plot experiment with three replications was conducted to evaluate the effects of sodium nitroprusside (SNP) application rate as an NO donor (0, 100, and 200 µM) on the growth and yield parameters of *Silybum marianum* L. (*S. marianum*) under different irrigation cut-off times (control, irrigation cut-off from stem elongation, and anthesis). The results of this study showed that with increasing drought severity, leaf RWC, proline content and capitula per plant, 1000 grain weight, plant height, branch per plant, capitula diameter, and the biological and grain yield of *S. marianum* decreased significantly, whereas the number of grains per capitula increased compared with the control. Also, by irrigation cut-off from the stem elongation stage, the density of leaf stomata at the bottom and top epidermis increased by 64% and 39%, respectively, and the length of the stomata at the bottom epidermis of the leaf decreased up to 28%. In contrast, the results of this experiment showed that the exogenous application of nitric oxide reduced the negative effects of irrigation cut-off, such that the application of 100 µM SNP enhanced RWC content (up to 9%), proline concentration (up to 40%), and grain (up to 34%) and biological (up to 44%) yields in plants under drought stress compared with non-application of SNP. The decrease in the number of capitula per plant and capitula diameter was also compensated by foliar application of 100 µM SNP under stress conditions. In addition, exogenous NO changed the behavior of the stomata during the period of dehydration, such that plants treated with SNP showed a decrease in the stomatal density of the leaf and an increase in the length of the stomata at the leaf bottom epidermis. These results indicate that SNP treatment, especially at 100 µM, was helpful in alleviating the deleterious effects of water deficiency and enhancing the tolerance of *S. marianum* to withholding irrigation times.

## 1. Introduction

Medicinal plants are valuable sources of pharmaceuticals. The milk thistle (*Silybum marianum* L. Gaertn.) has been a critical part of ancient medicine for over 2000 years and is an annual or biennial crop belonging to the Asteraceae family, which grows in many parts of the world, particularly in warm and dry regions [1]. *Silybum marianum* is a plant with low energy input for production, which is mainly cultivated as a medicinal plant, although it is also considered a food source [2]. The active ingredient in *S. marianum* is silymarin, a chemical extracted from the seeds that can protect the liver from toxins [3]. In addition, the fruits of this plant have 20–25% good quality oil; therefore, milk thistle is a multi-purpose crop that is used for agricultural production in marginal environments [4].

Owing to the limitation of water resources in most regions of the world, especially dry regions, the proper use of irrigation water and the cultivation of drought-resistant plants can play an important role in the continuation of plant production in arid and semi-arid regions [5]. Drought is one of the most important environmental factors limiting the growth and production of medicinal plants in many regions of the world [6,7,8,9,10,11,12]. Drought stress has deleterious effects on plant metabolic processes such as water relations, membrane damage, and assimilate partitioning [10,12,13,14]. The effects of water deficit usually manifest as a reduction in photosynthesis and plant growth [8,11,15,16]. Physiological parameters such as, growth rate, (relative water content) RWC, and stomatal conductance, show strong positive correlations with water use efficiency and with increasing water shortage, which also showed a sharp decrease [17]. In a study of drought stress on the milk thistle plant, the dry and fresh weight of the plant was significantly reduced at 50% water capacity treatment compared with that of well-watered plants [18]. Drought stress in oilseed rape decreased seed yield and oil content but increased unsaturated fatty acids in winter [19]. Severe drought stress significantly reduces morphological and physiological parameters in *Ranunculus asiaticus* [20]. Water deficit in wheat seedlings sharply reduced the leaf relative water content (RWC) but markedly increased the proline content [21]. The mechanisms underlying the water deficit responses of medicinal plants are extraordinarily complex and vary considerably with developmental stage, plant species, and duration of water stress [22]. Studies have shown that various farming conditions, such as water availability, can affect the levels of active biological components (silymarin) in *S. marianum* [23,24]. Although the secondary metabolite content in some medicinal plants may increase under drought stress, there is a tradeoff between increased concentrations of secondary metabolites in plants and a reduction in biomass yield due to drought conditions [5]. Therefore, one strategy for improving medicinal plant yield is to produce plants that are relatively tolerant to drought and that have greater production under drought stress. Currently, the application of Nitric Oxide has created the potential for the enhancement of drought stress tolerance in plants [25]. Nitric oxide (NO) is a small, fairly stable gas molecule that is soluble in water and lipids, and is considered an important biological messenger molecule in plants that diffuses through the membrane [26,27]. Nitric oxide participates in the regulation of many physiological processes in plants, such as germination stimulation, leaf expansion, metabolism, pathogenic factors [28], programmed cell death [29], photosynthesis, and flowering [30]. NO regulates and organizes reactive oxygen species by inducing transcriptional reactions involved in translocation, plant defense, signal transduction, and cell death [31,32]. The application of NO donors usually strengthens plants against future stress either by inducing the antioxidant machinery or by stimulating the production of endogenous NO [33]. The exogenous application of NO in *Dendrobium huoshanense* increased the RWC and antioxidant enzymes, but higher NO concentrations aggravated the effects of drought stress [29]. Drought stress seriously reduces hulless barley growth and physiological attributes, but NO alleviates the stress effects [27]. In some experiments that studied the effects of NO in rice, it was concluded that 100 µM SNP (sodium nitroprusside) had a greater influence on photosynthesis and seedling growth under stressful conditions [34,35]. Iran is located in an arid and semi-arid region with average annual precipitation, and because of low rainfall and limited water resources, the use of drought-adapted plants as well as growth stimulants to increase plant tolerance to drought can be a priority [36,37,38].

Thus, the objective of this study was to evaluate the effects of nitric oxide signaling on some developmental and functional parameters, as well as on the behavior of leaf stomata of *S. marianum* under water shortage conditions. In other words, the present study investigates the possible implications of foliar SNP treatment at various concentrations in promoting water deficit tolerance in milk thistle based on some morphological and physiological attributes with the aim of reducing irrigation water consumption in agriculture and increasing its cultivated area in arid and semi-arid regions.

## 2. Materials and Methods

### 2.1. Experimental Design and Treatments

The study was laid out as a split-plot arrangement in a randomized complete block design with three replications to evaluate changes in the growth and yield parameters of *S. marianum*. The present study was conducted in a research field located at the Faculty of Agriculture of Zanjan University, Zanjan, Iran, in 2020 (latitude: 36°: 41° N, longitude: 48°: 24° E, and altitude of 1620 m above sea level). The area is characterized as semiarid and has an annual record for a local temperature of 11.2 °C, including a maximum annual temperature of 16.5 °C, minimum annual temperature of 3.2 °C, and 285 mm annual prescription, on average. The soil in which the experiment was conducted possessed a clay loam texture with a pH of 7.85 (Table 1).

**Figure 1:**
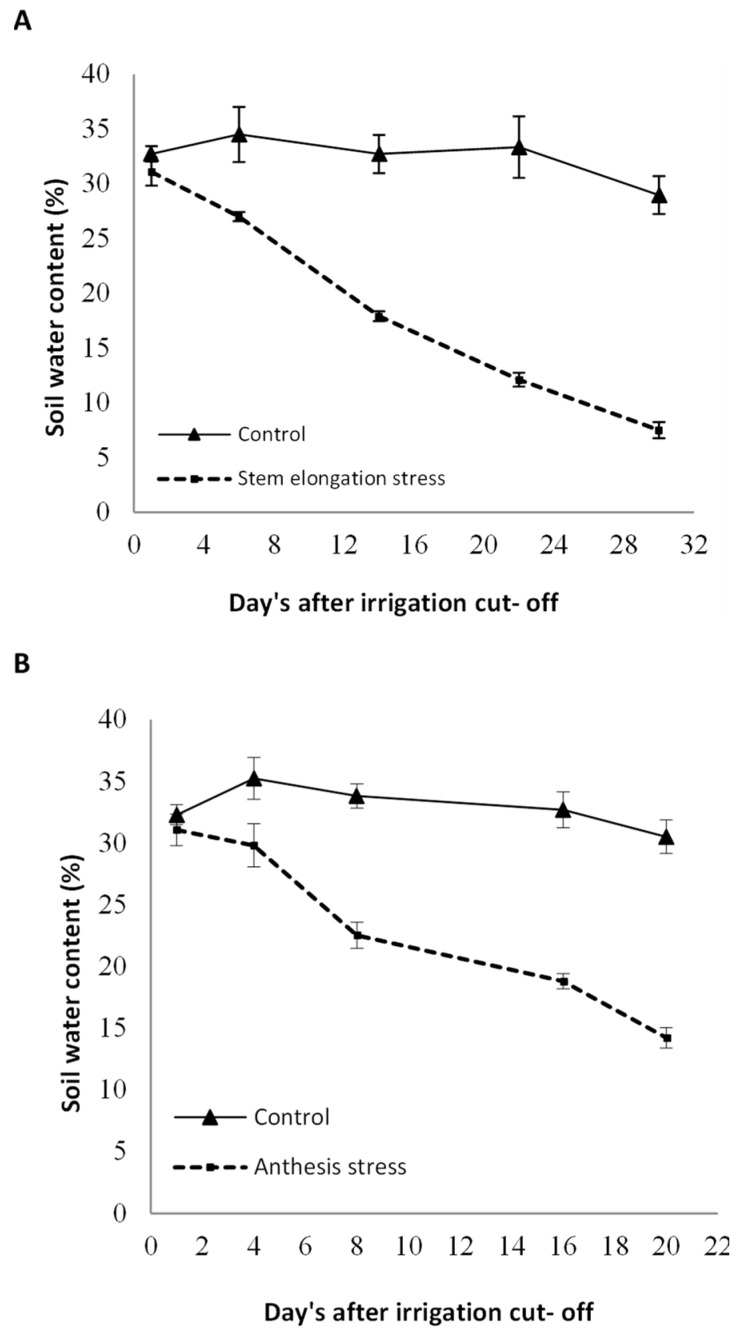
Soil–water content after irrigation cut-off beginning stem elongation stage (**A**) and anthesis (**B**) to the end of the growth period compared with control irrigation. Bars indicate the standard error.

Foliar spray with sodium nitroprusside as a Nitric Oxide donor at three levels of 0, 100, and 200 µmol L^−1^ was considered as the main factor, and the irrigation regime, including full watering (control, without irrigation cut-off to the end of the growth period), watering until mid-stem elongation (irrigation cut-off beginning stem elongation stage to harvest, on average, 60 days after sowing), and watering until anthesis (irrigation cut-off beginning anthesis stage to harvest, on average, 75 days after sowing) were allocated to the sub-factor. Irrigation was performed using irrigation tape, and the plants were watered every seven days until the start of withholding irrigation. After withholding the irrigation, no effective rainfall occurred at the experimental site. Milk thistle seeds comprised an indigenous landrace (Sari ecotype) from the Pakan Seed Company located in Isfahan. The seeds were sterilized using 0.2% carboxin thiram fungicide prior to sowing. Seeds were sown on 30 March 2020. Sub-plots comprised six 4-m rows spaced 50 m apart, with a density of 8 bushes per square meter. Fertilizer N was applied in irrigation water based on a soil test totaling almost 60 kg N ha^−1^ in split applications before cultivation and again before stem elongation. SNP was sprayed on the whole plant twice: after the rosette stage, at the flowering stage, and before and after sunrise. The volume of sprayed SNP for each treatment was 90 mL m^−2^. The SNP concentration was selected based on studies conducted by some researchers and in plants similar to milk thistle [27]. To evaluate the amount of moisture removed from the soil after stopping irrigation, the volumetric soil water content in several stages was measured at a depth of 0–30 cm using time-domain reflectometry TDR (model 60–50 × 1, ELE–Ltd., Leighton Buzzard, UK). The curves related to the soil moisture reduction are shown in Figure 1.

### 2.2. Relative Water Content (RWC)

The relative water content was determined according to the method described by Schonfeld et al. (1988) [39]. Relative water content (RWC) was measured 22 d after the irrigation cutoff from the stage of stem elongation. To determine RWC, fresh leaves were weighed immediately to obtain fresh weight (FW). Then, the leaves were soaked in deionized water for 6 h, saturated weight (SW) was determined, and the leaves were dried for 24 h at 80 °C to determine dry weight (DW). RWC was calculated as:

RWC (%) = (FW − DW)/(SW − DW) × 100

### 2.3. Determination of Free Proline

Leaf proline content was determined according to the method described by Bates et al. (1973) [40]. Fresh leaves (0.5 g) were homogenized in 5 mL of 3% sulfosalicylic acid solution and centrifuged for 15 min at 4000× *g*. Subsequently, 2 mL of the supernatant was mixed with 2 mL acid-ninhydrin and 2 mL of glacial acetic acid in a test tube. The mixture was then heated for 1 h at 95 °C in a water bath. After cooling the reaction mixture, 4 mL of toluene was added and shaken well, and the absorbance of the upper layer was measured at 520 nm using a UV/VIS spectrophotometer (PerkinElmer, Lambada 25, Waltham, MA, USA). Proline content was calculated using a calibration curve of the proline standard and expressed as µmol g^−1^ fresh weight.

### 2.4. Electrolytic Leakage

Measurement of electrolyte leakage was measured according to the method described by Lutts et al. (1996) [41]. Fresh leaf samples were cut to a certain level and incubated in test tubes containing 10 mL of double-distilled water at 25 °C for 24 h to measure the initial electrical conductivity (EC1) using a conductivity meter. The samples were then placed in a hot water bath at 100 °C for 35 min to measure the second electrical conductivity (EC2) after reaching room temperature. Electrolytic leakage was calculated as a percentage, using the following formula:

Electrolyte leakage = (EC1/EC2) × 100

### 2.5. Stomatal Characteristics

Leaf stomatal density, length, and width of the stomata at the top and bottom epidermis of the leaf were evaluated using the methods described by Radoglou and Jarvis (1990) [42] and Malone et al. (1993) [43]. Leaves from the same position were used for all treatments. The leaf surface was first wiped with a clean cotton pad, and then smeared with a thin layer of transparent liquid glue. The thin layer was separated from the leaf surface, fixed on a glass slide, and checked for the number of the stomata using an optical microscope (Leica Galen ΙΙΙ model, Hicksville, NY, USA). Stomatal density was expressed as the number of stomata per mm^2^. The length and width of stomata were measured as the length of the micrometer and expressed in µm. The microscopic images of the leaf stomata under stem elongation stress conditions and application of 100 µM SNP are shown in the Section 3.

### 2.6. Parameters of Yield Components

The number of capitulae per bush, plant height, and number of branches per plant were measured through a random selection of 20 plants in each plot. The number of grains per capitula, 1000-seed weight, and capitula diameter were obtained through the random selection of 50 capitula from each plot.

### 2.7. Grain and Biological Yield

After physiological maturity, the plants were hand-harvested from a 4 m^2^ in the central rows on 10 August 2020. Grain yield was determined because approximately 60% of the seeds had developed a pappus prior to seed dispersion. Grain and biological yields were reported in kg ha^−1^.

### 2.8. Statistical Analysis

Two-way ANOVA was used to detect differences between the deficit irrigation treatments and SNP application rates. The GLM procedure of the SAS statistical software (SAS, Version 9.1) was used for the analysis of variance, and means were compared using the least significant difference (LSD) test at *p* < 0.05. Correlation coefficients between yield and RWC and some stomatal traits whose variance analysis results were significantly determined through treatment means (*n* = 18) using SAS software. Finally, figures were drawn using Office 365.

## 3. Results

### 3.1. Relative Water Content (RWC)

The effects of drought stress and the interaction between SNP and drought stress on leaf relative water content were significant (Table 2).

**Figure 2:**
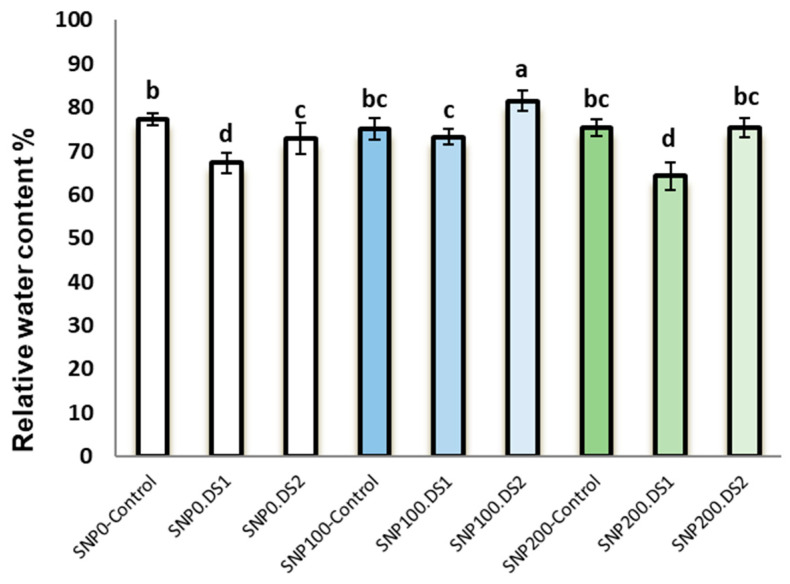
Effects of SNP application rates on milk thistle leaf RWC under deficit irrigation. Values are average three replications. Different letters on the top of the bars indicate significant difference at *p* < 0.05 by Duncan. Bars indicated standard error. Control: Well-watered; DS1: Drought stress in the stage of stem elongation and DS2: in the stage of anthesis.

Irrigation cut-off from the stages of stem elongation and anthesis significantly decreased leaf relative water content with a trend of decreasing soil moisture (Figure 1) compared with the control (Figure 1); however, this decline was significant with the application of 100 µM SNP, which led to a significant increase in leaf RWC compared with SNP non-application (Figure 2). Application of 200 µM SNP had no significant effect on the water status of plants.

### 3.2. Leaf Electrolyte Leakage

**Figure 3:**
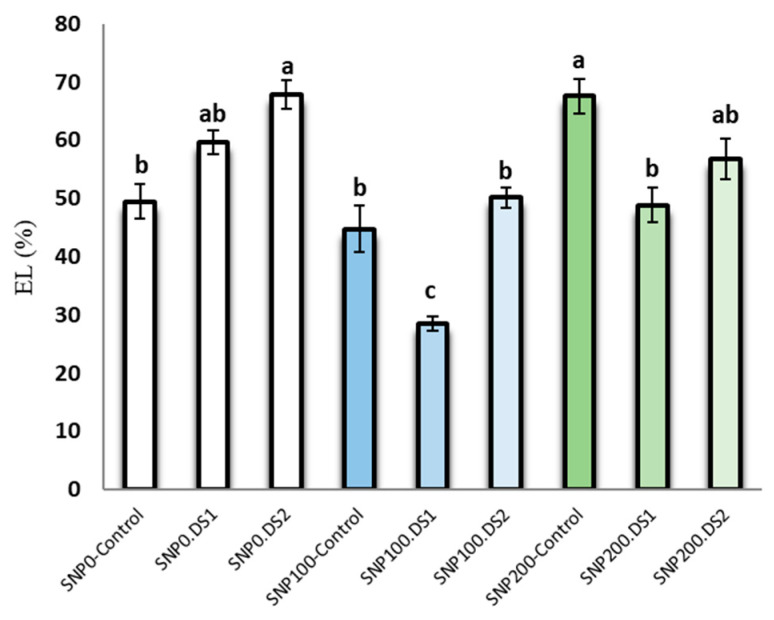
Effects of SNP application rates on *S. marianum* leaf electrolyte leakage percentage under deficit irrigation. Values are average three replications. Different letters on the top of the bars indicate significant difference at *p* < 0.05 by Duncan. Bars indicated standard error. Control: Well-watered; DS1: Drought stress in the stage of stem elongation and DS2: in the stage of anthesis.

The effects of SNP and irrigation cut-off and the interaction of SNP × irrigation cut-off were significant for leaf electrolyte leakage (Table 2). Electrolyte leakage in *S. marianum* leaves was increased by withholding irrigation (Figure 3). In contrast, exogenous application of 100 µM SNP significantly reduced electrolyte leakage by 50% and 24% from the stages of stem elongation and anthesis stress, respectively, compared to the non-application of SNP (Figure 3).

### 3.3. Free Proline Content

**Figure 4:**
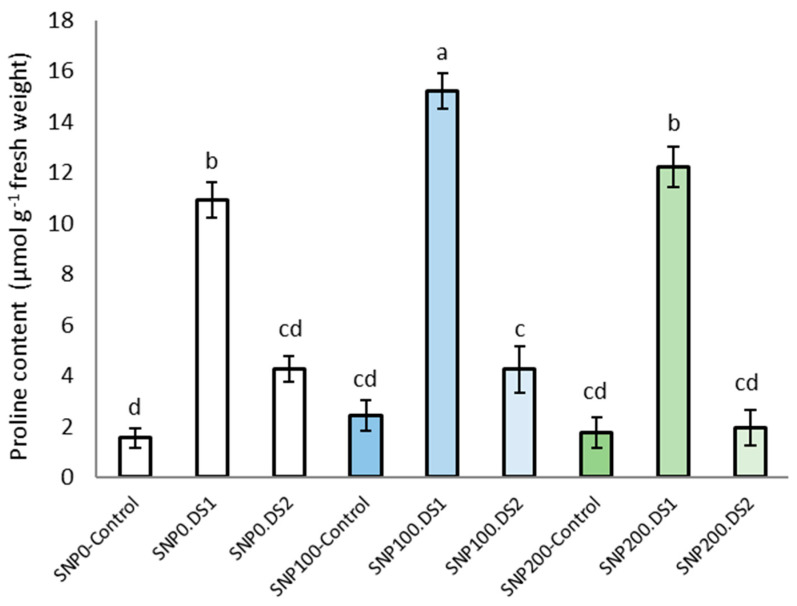
Effects of SNP application rates on *S. marianum* proline content under deficit irrigation. Values are average three replications. Different letters on the top of the bars indicate significant difference at *p* < 0.05 by Duncan. Bars indicated standard error. Control: Well-watered; DS1: Drought stress in the stage of stem elongation and DS2: in the stage of anthesis.

The effects of SNP and irrigation cutoff, and the interaction of SNP × irrigation cutoff, were significant for leaf proline content (Table 2). The maximum proline concentration was observed in plants under the irrigation cut-off from the stem elongation stage, but this increase was not significant from the anthesis stress stage (Figure 4). A further and significant increase in proline accumulation by 40% in plants under stem elongation stress was observed with the application of 100 μM SNP compared with water-stressed plants without SNP (Figure 4).

### 3.4. Stomatal Characteristics

**Figure 5:**
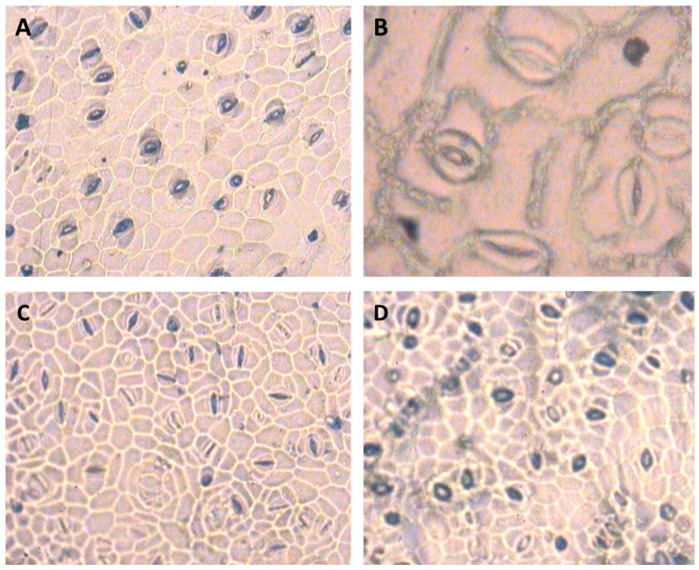
Microscopic images of milk thistle leaf stomata—(**A**) stomata of the lower epidermis of the leaf under normal conditions. (**B**) Detail of the stomata of the upper epidermis of the leaf under normal conditions. (**C**) stomata in the lower epidermis of the leaf under the influence of stem elongation stress. (**D**) Stomata in the lower leaf epidermis under the influence of stem elongation stress after application of 100 µM SNP. The image has a magnification of 40 micron with an area of 0.1589 mm^2^.

The ANOVA results of analysis of variance for stomatal traits are shown in Table 2. The results showed that the irrigation cut-off from the stem elongation stage significantly decreased stomatal length at the bottom epidermis of *S. marianum* leaves by up to 28% compared to the control plants (Table 3). However, the application of 100 µM SNP compensated for the reduction in stomatal length in the bottom epidermis under stress conditions (Table 3). The application of SNP significantly reduced stomatal width in the bottom epidermis under stress conditions compared to the non-application of water (Table 3). Under withholding irrigation from the stem elongation stage, stomatal density at the bottom and top epidermis of the leaf increased by 64% and 39%, respectively, compared to the control plants. However, plants treated with SNP under water deficit showed a decrease in stomatal density at the bottom (44%) and top (33%) epidermis of the leaf compared to the plants exposed to irrigation cut-off without SNP (Table 3). In addition, the irrigation cut-off from the anthesis stage had no significant effect on stomatal traits (Table 3). Among the stomatal traits, there was a negative and significant correlation between stomatal density at the bottom epidermis and stomatal length at the bottom epidermis, indicating a greater effect of the length of the stomatal apparatus than the width of the stomata in regulating the opening and closing of the stomata, especially under dry conditions (Table 4). Figure 5 shows representative images of the effects of the treatment on the distribution of stomata.

### 3.5. Yield Components

Capitula per plant, 1000 grain weight, plant height, grains per capitula, branch per plant, and capitula diameter were significantly affected by drought stress (Table 5). None of these traits was influenced by SNP application. The interaction of irrigation cut-off × SNP for these traits, except for the number of capitula per plant and capitula diameter, was also insignificant (Table 5).

Withholding irrigation due to stem elongation significantly decreased the number of capitula per plant, plant height, branch per plant, and capitula diameter compared with the control and anthesis stress; however, 1000 g weight showed the greatest significant decrease in both stages of stem elongation and anthesis stress, although the number of seeds increased (Table 6).

**Figure 6:**
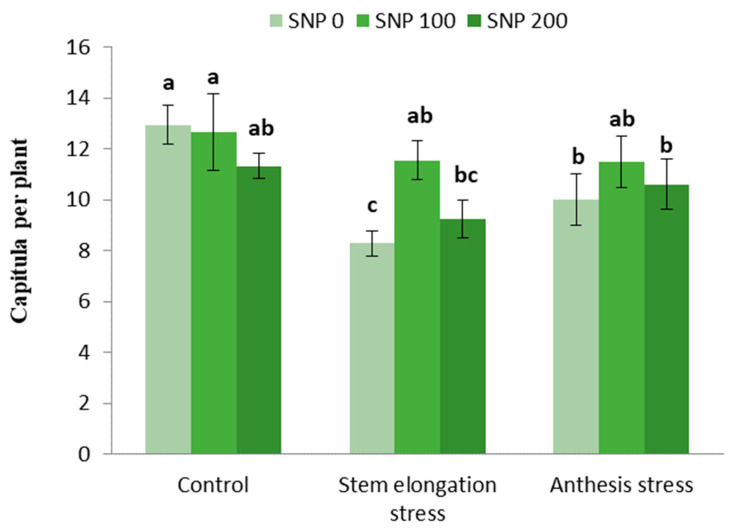
Effects of SNP application rates on the number of capitula per plant under deficit irrigation. Values are average three replications. Different letters on the top of the bars indicate significant difference at *p* < 0.05 by Duncan. Bars indicated standard error.

Irrigation cut-off at the stem elongation stage caused a 45% decrease in the number of capitula per plant compared with the control, whereas exogenous treatment with 100 µM SNP compensated for this decrease and caused a 44% increase in the number of capitula compared with non-application of SNP under stress conditions (Figure 6).

**Figure 7:**
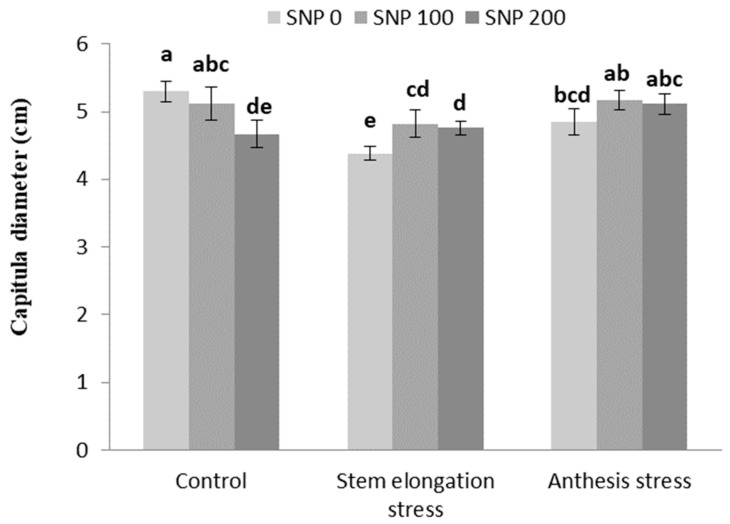
Effects of SNP application rates on *S. marianum* capitula diameter under deficit irrigation. Values are average three replications. Different letters on the top of the bars indicate significant difference at *p* < 0.05 by Duncan. Bars indicated standard error.

The number of grains per capitula increased with increasing drought severity at both stages of irrigation cut-off (Table 6). Foliar application of SNP led to an increase in capitula diameter under stem elongation stress compared to non-application (Figure 7).

### 3.6. Biological and Grain Yield

**Figure 8:**
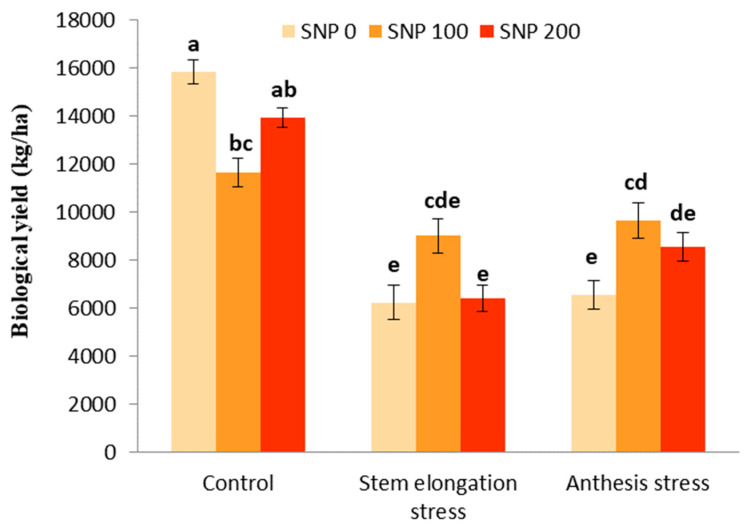
Effects of SNP application rates on *S. marianum* biological yield under deficit irrigation. Values are average three replications. Different letters on the top of the bars indicate significant difference at *p* < 0.05 by Duncan. Bars indicated standard error.

The effects of drought stress and the interaction between SNP and drought stress were significant for biological and grain yields (Table 5). However, SNP application had no significant effect on the biological yield (Table 5). With the trend of decreasing soil moisture (Figure 1), biological yield significantly decreased by 60% after the irrigation cut-off from the stages of stem elongation and anthesis (Figure 8). The exogenous application of 100 µM SNP only led to a significant increase in biological yield during anthesis stress, and stem elongation stress did not significantly increase the biological yield compared to its non-application (Figure 8).

**Figure 9:**
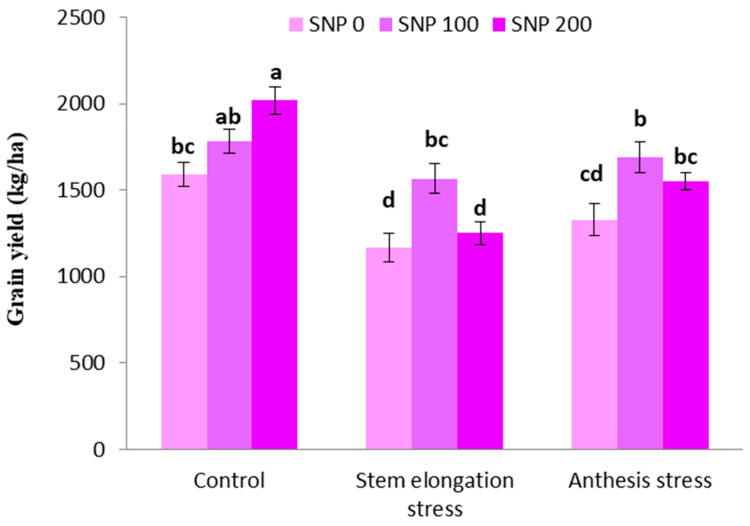
Effects of SNP application rates on *S. marianum* grain yield under deficit irrigation. Values are average three replications. Different letters on the top of the bars indicate significant difference at *p* < 0.05 by Duncan. Bars indicated standard error.

The highest grain yield was achieved in plants treated with 100 µM SNP under well irrigated conditions. The irrigation cutoff at the stem elongation stage significantly decreased grain yield, but this decline was insignificant at the anthesis stage (Figure 9). On the other hand, exogenous SNP application significantly compensated for the negative effects of grain yield decline in the period of drought; therefore, spraying with 100 µM SNP enhanced grain yield by 34% and 27% under severe and moderate drought stress, respectively, compared to plants treated merely under drought stress (Figure 9). However, the application of 200 µM SNP during both intervals of irrigation withholding did not have a significant effect on grain yield.

## 4. Discussion

**Figure 10:**
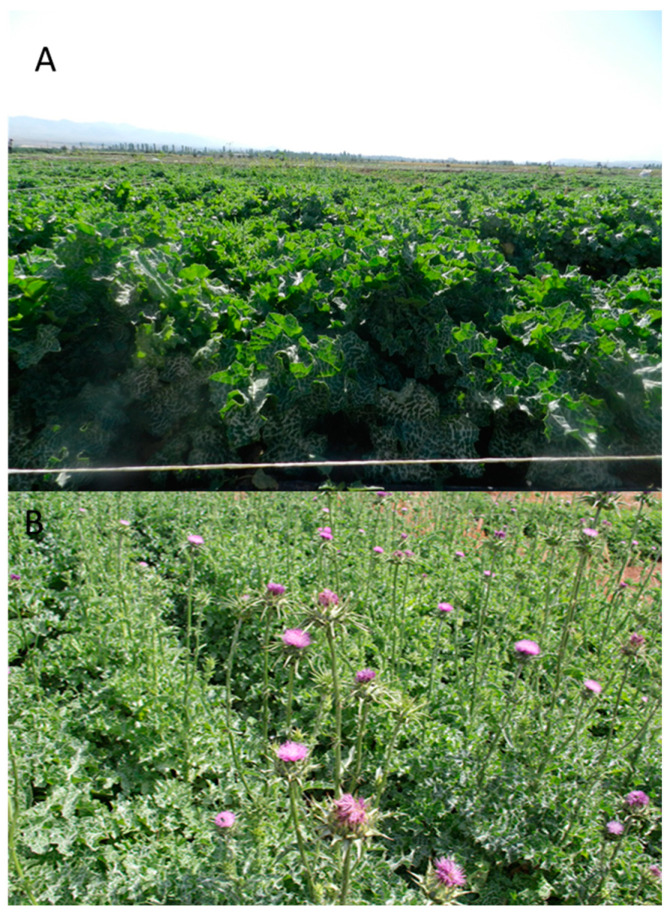
Milk thistle field grown in the research field at the rosette exit stage and the beginning of the stem elongation (**A**) and flowering stage (**B**).

In general, water shortages are a constraining factor in the production of many crops under farming conditions. Figure 10 shows a representative photograph of the experiment conducted in the open field on milk thistle crops from the rosette exit phase to the flowering phase. A decline in leaf relative water content and increased electrolyte leakage in *S. marianum* leaves were observed under drought stress; nonetheless, SNP application substantially improved leaf water status and cell membrane stability. A reduction in Leaf RWC and an increase in electrolyte leakage are general phenomena observed under drought stress [6,12,15,44,45]. In plant cells, ROS can cause oxidative damage and peroxidation of membrane lipids, resulting in leakage of cellular membranes, which damages photosynthesis and chloroplast pigments [46]. Some researchers too, in their studies concluded that by drought intensity escalation, leaf RWC significantly decreased [5,47]. Stressful conditions also significantly enhanced electrolyte leakage in *Ranunculus asiaticus* [20]. High levels of accumulated proline and pigments under stress conditions enable some plants to maintain turgor and, thus, water potential [34]. Under conditions of water deficit, the amount of water in leaf tissues and cells decreases due to low osmotic potential. As a result, RWC also decreases [48]. Under water stress, RWC was increased by the application of 100 μM SNP. Applying signal molecules, such as nitric oxide (NO) donors, can improve plant resistance to environmental stress. Application of SNP increased the accumulation of osmotic regulators (e.g., proline), which decreased osmotic potential and led to maintenance and increased water uptake, and consequently RWC [49,50]. Exogenous SNP improve drought tolerance in maize, rice, and barley by increasing cell membrane stability and RWC [27,51]. Furthermore, Application of the SNP by increasing the glyoxalase system enzymes restored the leaf RWC and further increased the proline content under water stress conditions [21].

Similarly, a water deficit in soybean plants treated with melatonin alleviated oxidative damage in leaves via a reduction in electrolyte leakage levels [52]. In the present study, 100 µM exogenous SNP significantly preserved the cell membrane against oxidative stress during drought conditions.

Proline accumulation occurs in a wide range of plant species in response to various environmental stresses such as drought. In this study, proline accumulation increased significantly with increasing water deficit. Increased proline content in response to water stress has been reported in several plants [18,53,54]. Under stress conditions, one mechanism for osmotic adjustment is the accumulation of compatible solutes such as the amino acid proline. Proline is a non-enzymatic antioxidant metabolite used for energy storage under drought conditions, resulting in water-deficit tolerance and ROS-scavenging ability [55]. Moreover, with the application of 100 µM SNP under withholding irrigation, more proline accumulated in the leaves compared to no SNP application. It was concluded that the accumulation of proline can be induced by the application of NO, which can activate some key enzymes in the synthesis of proline and, as a result, increase it [46,56]. Recently, it has been claimed that exogenous NO can induce the P5CS1 gene encoding 1-pyrroline-5carboxylate synthetase, a key enzyme involved in proline synthesis [57]. Moreover, NO is known to act as an antioxidant [29]; therefore, it prevents proline breakdown. Similar results have been reported for rice plant [58].

The results of this experiment showed that the irrigation cut-off from the stem elongation stage affected the number of stomata per unit area more than closing the stomata to increase stomatal resistance (Table 3). In addition, these results indicated that the length of the stomata was affected and decreased during the closing of the stomata under stress conditions (Table 3). In contrast, the application of 100 µM SNP prevented the increase in stomatal density and the closing of more stomatal pores under water deficit conditions (Table 3). Stomata respond to environmental conditions such as stress, and their number and distribution may change [59]. SNP applications can play a physiological role in stomatal movement [60]. Most studies have demonstrated the ability of NO to reduce the stomatal aperture [33,61], which is involved in gas exchange. Thus, the maximum stomatal conductance can be determined by the morphological characteristics of the stomata, such as stomatal size and density [62]. Therefore, although NO has been demonstrated only in relation to the control of stomatal opening and closure [63], the results of this study showed that NO is involved in the development and formation of guard cells under water deficit conditions and, therefore, may affect stomatal distribution in the leaf or conserve leaf water as an increment in foliar trichomes [64]. In addition, studies have shown that exogenous NO releases Ca^+2^ in guard cells, which regulates K^+^ and Cl^−^ channels in the plasma membrane and induces stomatal closure [50,65]. These results are in agreement with those of studies conducted on Arabidopsis [60] and soybean [49]. It has been reported that in bean plants under salinity stress, the size of stomata decreased with CCC application, but the number of stomata increased per unit of leaf area [66]. Stomatal density and length at the bottom epidermis showed significant positive and negative correlations with RWC, respectively. In addition, among the stomatal traits, only the lm between stomatal density at the bottom epidermis and grain yield was positive and significant (Table 4). It seems that under conditions of drought and SNP application, although the number of stomata in the leaf increased due to the reduction of the leaf surface, the leaves prevented more water loss by adjusting the length of the stomata, thus increasing the plant’s tolerance to water deficit. The results of some researchers have also confirmed this [64].

Reduction in capitula per plant, grain weight, plant height, branch per plant, and diameter of capitula under stress conditions (Table 6) are likely related to a decline in cell division and expansion, photosynthesis, nutritional imbalances, and disruption of the absorption and distribution of water and nutrition [67,68]. Drought stress decreases the grain-filling period, and as a result 1000 grain weight is reduced. Plant height decline in response to drought may be due to a decrease in turgor, cell elongation, cell growth [69], and xylem and phloem vessel blockage, and hence the prevention of any transfer in this manner [70]. A decline in the yield components and plant height of *S. marianum* under irrigation treatments has been reported previously [71,72]. In the present study, the number of grains per capitula increased under drought stress conditions (Table 6). Drought stress caused a 45% decrease in the number of capitula per plant and a 7% decrease in the diameter of capitula compared with the control. This decline in the number of capitula per plant was greater than that in capitula (Table 6). Therefore, larger capitula per plant along with smaller seeds were produced under drought stress (Table 6); hence, the increase in the number of grains per capitula under water shortage conditions seems reasonable.

Improving the diameter of the capitula in response to 100 µM SNP treatment under stem elongation stress could be the result of enhanced plant growth rate, regulation of processes such as leaf expansion and vegetative growth, and increased accumulation of dry matter [73].

The biological and grain yields of *S. marianum* were negatively affected by the irrigation cutoff. Water deficit causes a series of physiological, morphological, and biochemical changes in plants, such as reduction in growth and photosynthesis limitations [25,54]. Photosynthetic limitation caused by water deficiency, especially in plants under severe drought conditions, results in lower biomass accumulation. Thus, photosynthesis assimilates transferred to the grain are reduced [35,74], which decreases the ultimate plant yield under farming conditions. In addition, leaf area decreases under stress conditions, thus reducing carbon assimilation by degrading the pigments used in photosynthesis and negatively impacting plant yield and growth [75]. Therefore, with increasing water deficit, *marianum* grain yield declines [5,71]. It has been reported that water deficit at the growth stages and for longer than 12 d at seed filling is the most effective factor in reducing grain yield in parsley [54,76]. The exogenous application of SNP positively affected *S. marianum* grain yield under drought stress. Sodium nitroprusside, specifically at a concentration of 100 µM, caused a reduction in lipid peroxidation and consequently increased water use efficiency and accumulation of dry material. This was achieved by reducing stomatal conductance and transpiration, decreasing ROS, particularly H_2_O_2_ and increasing antioxidant enzyme activity [5,71]. Moreover, exogenous SNP application can improve the fluidity of the membrane, especially the phospholipid bilayer, enhance cell membrane stability, photosynthesis, and water status of the leaf, and finally enhance plant growth [34]. The transfer of more assimilates to the grain results in the enhancement of grain yield under stress [35,77]. The enhancement in growth and yield due to SNP application under drought stress is due to the preservation of relative water content and the reduction of reactive oxygen species [47,78]. In other words, it has been demonstrated that the positive impact of SNP at a lower concentration on biological yield might be due to an increase in photosynthesis by increasing chlorophyll content [5,71], a decrease in oxidative damage by reducing H_2_O_2_ [5,71], the protection of cell membranes from cellular peroxidation [79,80], and the potential NO in cytokinin signaling in the plant [81]. Some researchers also reported that under water deficit, spraying 1mM of Salicylic acid in *Allium hirtifolium* had more significant effects on higher seed yield, compared to 0.5 Mm [5,71].

It should be noted that a better understanding of the impact of SNP in mitigating the effects of drought stress in milk thistle requires a study of its molecular mechanisms. Further studies using different SNP concentrations are warranted to achieve maximum water deficit tolerance.

## 5. Conclusions

This study revealed that NO-induced improvement in water deficit tolerance in milk thistle by reducing membrane leakage, increasing proline and RWC, and regulating stomatal behavior under stress conditions. *S. marianum* plants under stem elongation and anthesis drought stress exhibited 27 and 16% lower grain yield, respectively, and 60% lower biological yield than well-watered plants. In this study, RWC, proline content, stomatal status, and yield parameters except for 1000 grain weight were only affected by long-term drought stress, and the stress from the anthesis stage did not have a significant effect on some of these traits. Among the yield components, the number of capitula per plant was the most sensitive to deficit irrigation, followed by the 1000 grain weight. According to the results obtained, exogenous SNP significantly decreased electrolyte leakage and increased RWC, proline content, capitula diameter, and biological and grain yield under water deficit conditions and compensated for the reduction in negative effects at the stages of stem elongation and anthesis stress. Furthermore, 100 µM SNP was more efficient than 200 µM SNP in improving *marianum* growth and yield of *S. marianum* under drought stress. Considering the importance of the milk thistle plant in the pharmaceutical and oil extraction industries, increasing the cultivated areas in arid and semi-arid areas should be considered. Therefore, the use of growth-stimulating signals such as SNP can be effective in increasing plant tolerance in these areas, thus increasing the yield of oil and active ingredients.

## Figures and Tables

**Table 1:** Some physical and chemical characteristics of the studied soil.

Potassium mg kg^−1^	Phosphorous mg kg^−1^	(EC) dS m^−1^	Sand (%)	Silt (%)	Clay (%)	Soil Texture	Organic Carbon (%)	(pH)
280	55.12	4.13	38.16	32	29.84	(Silty clay loam)	1.0	7.69

**Table 2:** Analysis of variance for RWC, proline content, electrolyte leakage and stomatal characteristics affected by SNP and drought stress.

S.O.V	df					Mean of Squares				
		RWC	Proline Content	Electrolyte Leakage	Stomatal Density at the Bottom Epidermis	Stomatal Density at the Top Epidermis	Stomatal Width at the Bottom Epidermis	Stomatal Width at the Top Epidermis	Stomatal Length at the Bottom Epidermis	Stomatal Length at the Top Epidermis
Replication	2	18.69	2.83	34.46	869.3	1335.2	3.93	1.43	2.27	1.7
Sodium nitroprusside (S)	2	34.99 ^n.s.^	53.97 **	415.7 *	4056.6 *	1011.7 ^n.s.^	21.7 **	1.67 ^n.s.^	3.85 ^n.s.^	11.3 ^n.s.^
Main plot error	4	17.08	3.39	241.8	2297.7	1234.2	8.4	6.79	18.2	7.5
Drought stress (S)	2	366.69 **	274.70 **	702.1	2317.7 *	178.3 ^n.s.^	1.39 ^n.s.^	3.58 ^n.s.^	35.2 **	8.9 ^n.s.^
Interaction SNP × S	4	88.32 **	18.74 *	278.4 *	2671.1 **	823.7 ^n.s.^	8.57 **	13.6 *	25.4 **	7.1 ^n.s.^
Sub plot error	12	12.22	6.21	81.5	732.3	567.6	2.5	3.8	5.3	12.9
Total	26									

^n.s.^, not significant, * Significant at *p* ≤ 0.05. ** Significant at *p* ≤ 0.01. SNP, sodium nitroprusside.

**Table 3:** Effects of SNP application on stomatal characteristics of milk thistle under deficit irrigation.

Deficit Irrigation	SNP (µM)	Stomatal Density at the Bottom Epidermis (Number mm^−2^)	Stomatal Density at the Top Epidermis (Number mm^−2^)	Stomatal Width at the Bottom Epidermis (µm)	Stomatal Width at the Top Epidermis (µm)	Stomatal Length at the Bottom Epidermis (µm)	Stomatal Length at the Top Epidermis (µm)
	0	137.9 ^b^	90.5 ^ab^	20.2 ^bc^	24.4 ^ab^	35.5 ^a^	41.5 ^a^
Control	100	143.7 ^b^	88.5 ^b^	19.2 ^c^	23.8 ^ab^	34.1 ^ab^	37.8 ^ab^
	200	139.3 ^b^	87.7 ^b^	19.3 ^c^	23.5 ^ab^	32.2 ^ab^	37.5 ^ab^
	0	226.5 ^a^	125.8 ^a^	22.3 ^ab^	20.5 ^b^	25.6 ^c^	36.9 ^ab^
stem elongation stress	100	127.7 ^b^	84.4 ^b^	19.3 ^c^	25.3 ^a^	31.8 ^b^	35.3 ^b^
	200	148.9 ^b^	82.5 ^b^	18.7 ^c^	22.1 ^b^	30.7 ^b^	38.9 ^ab^
	0	151.1 ^b^	97.2 ^ab^	21.5 ^b^	21.7 ^b^	30.9 ^b^	38.5 ^ab^
anthesis stress	100	117.1 ^b^	77.1 ^b^	21.1 ^b^	218 ^b^	31.1 ^b^	37.2 ^ab^
	200	154.1 ^b^	110.6 ^ab^	24.4 ^a^	26.1 ^a^	30.9 ^b^	36.9 ^ab^
	LSD	59.64	34.87	2.28	3.22	3.59	4.62

Results are the mean of three replications. Data presented from the two-way factorial scheme according to the significance of ANOVA. Means with one similar letter in each column have no signification difference at 5% of probability level (*p* < 0.05) by Duncan’s multiple range test. LSD, least significant difference (*p* < 0.05).

**Table 4:** Pearson’s correlation coefficients between yield and RWC and stomatal traits on milk thistle under irrigation cut-off and SNP application (*n* = 18).

Trait	1	2	3	4	5	6
Stomatal density at the bottom epidermis (1)	1					
Stomatal width at the bottom epidermis (2)	n.s.	1				
Stomatal width at the top epidermis (3)	n.s.	n.s.	1			
Stomatal length at the bottom epidermis (4)	−0.75 **	n.s.	n.s.	1		
RWC (5)	−0.935 **	n.s.	n.s.	0.52 *	1	
Grain yield (6)	0.52 *	n.s.	n.s.	n.s.	0.57 *	1

^n.s.^, not significant, * Significant at *p* ≤ 0.05. ** Significant at *p* ≤ 0.01. SNP, sodium nitroprusside.

**Table 5:** Analysis of variance for yield parameters and biological and grain yield affected by SNP and drought stress.

S.O.V	df				Mean of Squares				
		Plant Height	1000 GrainWeight	Grains per Capitula	Capitula per Plant	Branch per Plant	Capitula Diameter	Biological Yield	Grain Yield
Replication	2	1069.02	3.18	340.4	13.61	2.59	0.03	2,408,973	106,551.7
Sodium nitroprusside (S)	2	1077.7 ^n.s.^	3.9 ^n.s^	770.9 ^n.s^	15.91 ^n.s.^	0.52 ^n.s.^	0.48 ^n.s.^	1,259,827 ^n.s.^	378,050.6 *
Main plot error	4	631.4	1.1	471.54	7.06	0.28	0.57	6,175,478	15,598.5
Drought stress (S)	2	697.7 **	14.9 **	1090.3 **	102.3 **	0.82 **	14.72 **	57,925,097 **	795,510.1 **
Interaction SNP × S	4	67.7 ^n.s^	1.5 ^n.s.^	397.9 ^n.s.^	5.06 *	0.12 ^n.s.^	1.46 *	9,953,187 **	60,213.1 **
Sub plot error	12	80.8	0.8	169.5	1.51	0.09	0.23	1,882,326	10,409.6
Total	26								

^n.s.^, not significant, * Significant at *p* ≤ 0.05. ** Significant at *p* ≤ 0.01. SNP, sodium nitroprusside.

**Table 6:** Means of yield components of milk thistle affected of withholding irrigation times.

Treatments	Plant Height (cm)	1000 Grain Weight (g)	Grains per Capitula	Capitula per Plant	Branch per Plant	Capitula Diameter (cm)
Normal irrigation	130.97 ^a^	22.44 ^a^	75.75 ^c^	9.98 ^a^	5.71 ^a^	5.04 ^a^
Stem elongation stress	96.61 ^b^	20.09 ^b^	106.16 ^a^	5.49 ^b^	3.87 ^b^	4.65 ^b^
Anthesis stress	128.16 ^a^	20.78 ^b^	90.25 ^b^	8.54 ^a^	5.33 ^a^	5.03 ^a^

The results are presented as the mean of three replicates. Data are presented using a two-way factorial scheme according to the significance of the ANOVA. Means with one similar letter in each column are not significantly different at the 5% probability level (*p* < 0.05), according to Duncan’s multiple range test.

## Data Availability

Data are available from the corresponding authors upon request.
